# Fatty Acid Metabolic Signaling Pathway Alternation Predict Prognosis of Immune Checkpoint Inhibitors in Glioblastoma

**DOI:** 10.3389/fimmu.2022.819515

**Published:** 2022-02-18

**Authors:** Rongrong Liu, Weidong Liang, Qian Hua, Longqiu Wu, Xiangcai Wang, Qiang Li, Fangjun Zhong, Bin Li, Zhengang Qiu

**Affiliations:** ^1^ Department of Oncology, The First Affiliated Hospital of Gannan Medical University, Ganzhou, China; ^2^ Department of Neurology, Ganzhou People’s Hospital, Ganzhou, China; ^3^ Department of Neurology, The Second Affiliated Hospital of Nanchang University, Nanchang, China; ^4^ Department of Neurology, The First Affiliated Hospital of Gannan Medical University, Ganzhou, China; ^5^ Department of Anesthesiology, The First Affiliated Hospital of Gannan Medical University, Ganzhou, China; ^6^ Department of Emergency, The First Affiliated Hospital of Gannan Medical University, Ganzhou, China

**Keywords:** glioblastoma, immune-checkpoint inhibitors, immunotherapy, prognosis, fatty acid metabolic process

## Abstract

**Introduction:**

Glioblastoma(GBM) is a highly malignant primary brain tumor. Even after undergoing surgery and chemotherapy, patients with this affliction still have little to no chance of survival. Current research on immunotherapy treatment for GBM shows that immune-checkpoint inhibitors (ICIs) may be a promising new treatment method. However, at present, the relationship between the fatty acid metabolic process and the prognosis of GBM patients who are receiving immunotherapy is not clear.

**Methods:**

First, we downloaded a GBM cohort that had been treated with immunotherapy, which included the mutation and prognosis data, and the TCGA-GBM and Jonsson-GBM queues. CIBERSORT and single sample gene set enrichment analysis(ssGSEA) were used to evaluate immune cell scores. Gene set enrichment analysis (GSEA) was used to evaluate the patient’s accessment score. The pRRophetic algorithm was used to evaluate the drug sensitivity of each patient. Univariable and multivariate cox regression analyses, as well as the Kaplan-Meier (KM) method, were used to evaluate the relationship between the fatty acid metabolic process and the prognosis of GBM patients.

**Results:**

The univariate and multivariate cox regression models showed that the fatty acid metabolic process mutant-type (MT) can be used as an independent predictor of the efficacy of immunotherapy for GBM patients. In addition, fatty acid metabolic process MT is related with significantly longer overall survival (OS) time than the wild-type(WT) variant. However, the mutation status of the fatty acid metabolic process has nothing to do with the prognosis of GBM patients who are receiving conventional treatment. Our analysis showed that fatty acid metabolic process MT correlated with significantly increased natural killer T (NKT) cells and significantly decreased CD8+T cells. At the same time, GSEA analysis revealed that fatty acid metabolic process MT was associated with significantly increased immune activation pathways and an enriched fraction of cytokine secretion compared with WT.

**Conclusions:**

We found that fatty acid metabolic process MT may be used as an independent predictor of the efficacy of ICI treatment in GBM patients. Use of the fatty acid metabolic process MT will result in higher immunogenicity rates, a significant increase in the proportion of activated immune cells, and improvement of the immune microenvironment.

## Introduction

Even if a glioblastoma (GBM), a primary brain tumor with the highest degree of intracranial malignancy, can be safely and fully resected, the median survival time for patients is still only 14 month, and there is recurrence rate of 90% (1, 2). That being said, the main treatment of early GBM is still surgical resection. Radiotherapy and chemotherapy are also important methods for treating GBM, and some clinical studies have shown that these treatments can play an active role in improving the survival time of patients. However, these methods are not without their drawbacks (3, 4). Normal brain tissue damage is caused by the low selectivity of radiotherapy and chemotherapy, while the use of chemotherapy drugs can cause systemic immunosuppression. In addition, because of the complexity and dynamic changes of tumor microenvironments, chemotherapy drugs are prone to drug resistance (4–7).

In recent years, there has been a succession of breakthroughs in the field of tumor immunotherapy. During this time, the Food and Drug Administration (FDA) has approved a number of new immunotherapy drugs for various types of tumor therapies, such as immune-checkpoint inhibitors (ICIs) and chimeric antigen receptor (CAR) T-cells (8). At present, there are many studies exploring the application effect of GBM immunotherapy. These studies have shown that in the GBM mouse model, the therapeutic inhibition of IDO, cytotoxic T lymphocyte antigen-4 (CTLA-4), or programmed death-ligand 1 (PD-L1) can significantly reduce the number of Treg cells infiltrated by tumors while significantly increasing the long-term survival rate of patients (9). Research thus far suggests that ICIs may be a promising new immunotherapy treatment method for GBM (10–12). However, the complex inhibitory immune microenvironment and the gradual formation of cancer cells during the growth of GBM severely limit the treatment’s curative effect (13). Therefore, clarifying the mechanisms behind the various aspects of the immune microenvironment would provide an important theoretical basis for better understanding the internal causes of GBM’s malignant progression while also improving GBM immunotherapy strategies (14).

In recent years, studies have shown that treatment using fatty acid metabolism can change the inflammatory microenvironment and promote the progress and metastasis of tumors (15). This interaction constitutes an intricate metabolic network in which the lipid metabolism pattern of M2 macrophages is activated by tumor cells. Zhang et al. found that M2 macrophages depend on fatty acid oxidation (FAO) pathways to obtain energy. At the same time, they also promote the secretion of IL-1β by activating reactive oxygen species and NLRP3 inflammatory corpuscles, while also accelerating the proliferation, migration, and invasion of tumor cells (16). Wu et al. found that unsaturated fatty acid derived from lipid droplets in tumor cells induced myeloid cells to polarize to M2 macrophages by promoting mitochondrial respiration. This process ultimately leads to the failure of immune cell monitoring and increased tumor growth and metastasis (17). However, despite our increasing knowledge on the subject, the role of fatty acid metabolism in the prognosis of immunotherapy with GBM is still unclear. Therefore, the purpose of this study is to explore the relationship between fatty acid metabolic pathway and the prognosis of GBM immunotherapy and condition of the immune microenvironment, in order to screen out the dominant GBM population receiving immunotherapy and further improve the clinical benefits of GBM patients.

## Methods

### GBM Queue

Samstein et al. published a GBM queue (ICI-GBM) that accepts ICIs, including mutation data analyzed *via* targeted sequencing and survival data (18). Due to the limited number of GBM queues for immune checkpoint treatment, we also collected TCGA-GBM queues (19) from the GDC database and a Jonsson-GBM queue (20) from cBioPortal. The TGCA-GBM queues included expression data, mutation data, and survival data (20). The Jonsson-GBM queue included mutation data and survival data. See **Table S1** for details on the clinical features of patients in the ICI-GBM cohort.

### Mutation State of the Fatty Acid Metabolic Process Pathway

In order to explore the relationship between the mutation status of the fatty acid metabolic process pathway and the clinical prognosis of GBM patients after ICI treatment, we downloaded the gene set related to the fatty acid metabolic process from the Molecular Signatures Database (MsigDB) (https://www.gsea-msigdb.org/gsea/msigdb/genesets.jsp) (21) database in the format of a GMT file. First, we screened the mutation data in the ICI-GBM, TCGA-GBMs, and Jonsson-GBM queues based on the definition of non-synonymous mutation types in the maftools R package (22). After this initial screening, only non-synonymous mutation data was retained for subsequent analysis. From here, we calculated the mutation rate of each patient’s fatty acid metabolic process and defined the mutation state of each patient’s pathway according to whether any mutation occurred. If the number of mutations in this pathway was zero, it was considered a WT. If the number of mutations in this pathway was greater than 0, it was considered an MT.

### Immune Microenvironment Analysis and Drug Sensitivity Analysis

In order to explore the difference in the immune cell scores between the WT and MT groups, we used CIBERSORT and IOBR to evaluate the immune cell scores of each patient (23). In addition, we used ssGSEA and GSEA algorithms to evaluate the activation degree of patients in each pathway (24, 25). The emapplot function was used in our enrichment analysis to analyze the relationship between the pathways (26). We used the pRRophetic algorithm in our estimation of drug sensitivity to analyze the expression data of each patient (27). From this, we obtained the estimated IC50 value of each patient for 138 drugs from the Genomics of Drug Sensitivity in Cancer (GDSC) database (https://www.cancerrxgene.org/) (27).

### Statistical Analysis

**Figure 1:**
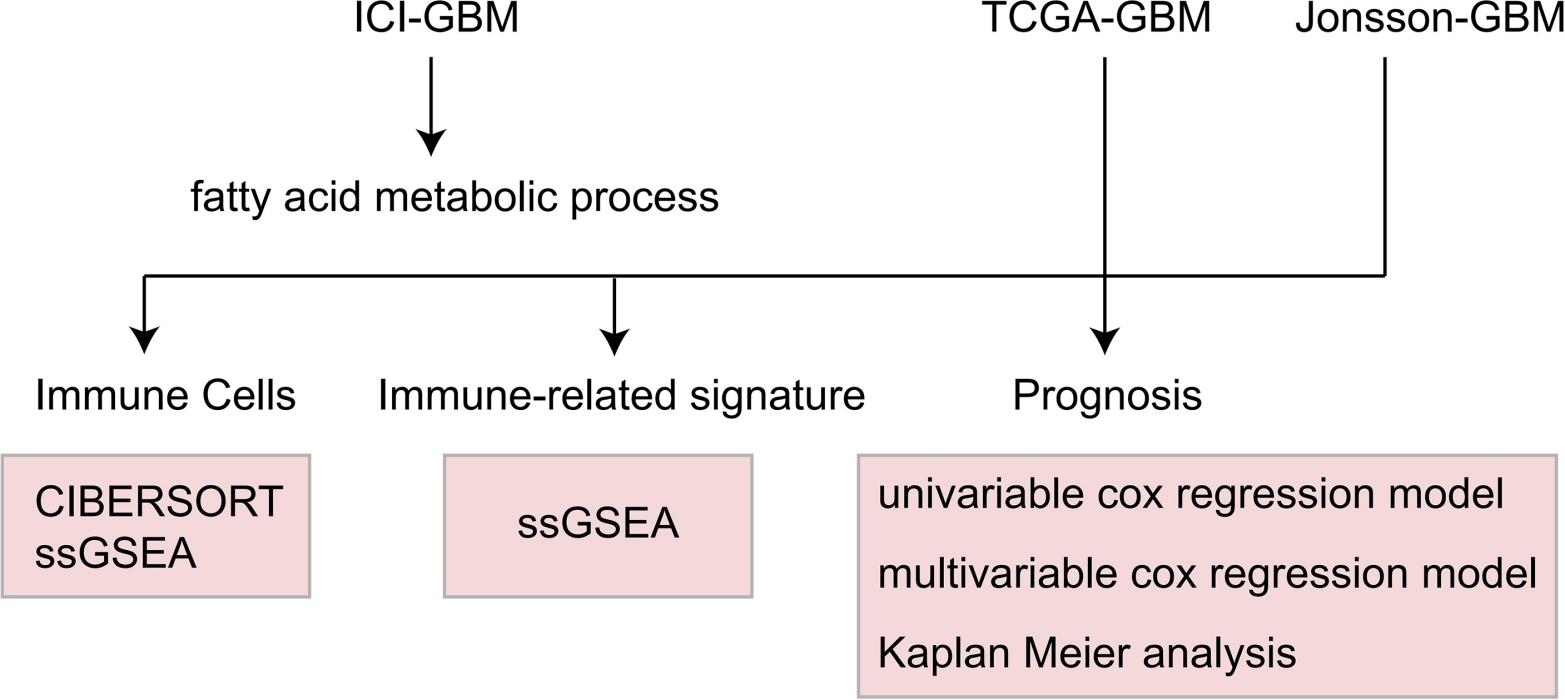
An overview of this study.

To explore the relationship between the fatty acid metabolic process pathway and the immunotherapy prognosis of GBM patients, we used Kaplan Meier (KM) survival analysis, univariate and multivariate cox regression analysis, and log-rank P to show the degree of statistical difference. In the difference analysis of continuous variables, the Wilcoxon-Mann-Whitney test was used to compare the amount of tumor mutation burdens (TMB), DNA damage repair (DDR) pathway mutations, and the immune cell scores. To estimate the difference of IC50 between the WT and MT groups, Fisher’s exact test was used to count the difference in the mutation frequency between each group. The difference was analyzed using the Limma Package (28). GSEA and enrichment analysis were performed using Clusterprofiler (26). A heat map was drawn using the Complexheatmap package (29). Box drawings were created using ggplot2 (30). The judging range of differential genes in the analysis was |log2FC|>1 and P < 0.05. The analysis flow of this study is shown in **Figure 1**.

## Results

### Prognostic Role of the Mutation State of the Fatty Acid Metabolic Process in GBM Treatment

**Figure 2:**
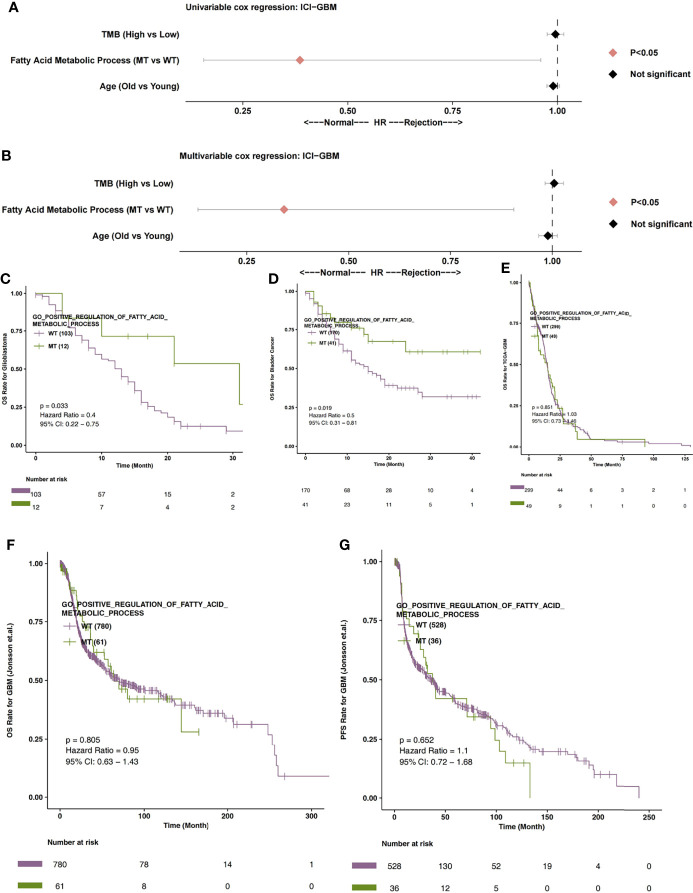
**(A)** The univariable Cox regression analysis model of ICI-GBM. **(B)** The multivariable Cox regression analysis model of ICI-GBM. **(C)** The Kaplan Meier curve of the fatty acid metabolic processes of the MT and WT groups in the ICI-GBM queues. **(D)** The Kaplan Meier curve of the fatty acid metabolic processes of the MT and WT groups in the ICI-BLCA queues. **(E)** The Kaplan Meier curve of the fatty acid metabolic processes of the MT and WT groups in the TCGA-GBM queues. **(F)** The Kaplan Meier curve of the fatty acid metabolic processes of the MT and WT groups in the Jonsson-GBM queues in relation to OS. **(G)** The Kaplan Meier curve of the fatty acid metabolic processes of the MT and WT groups in the Jonsson-GBM queues in relation to PFS. MT, mutant-type; WT, wild-type; OS, Overall Survival; PFS, Progression-free Survival.

In order to explore the relationship between the fatty acid metabolic process and the immunotherapy prognosis of GBM patients, univariate and multivariate cox regression analysis were applied to the ICI-GBM cohort. Univariate results showed that fatty acid metabolic process MT was related to significantly prolonged OS time in GBM patients (**Figure 2A**; P < 0.05; HR = 0.39). Through our analysis, we found that fatty acid metabolic process MT can be used as an independent predictor of ICIs in GBM patients (**Figure 2B**; P < 0.05; HR = 0.34). In ICI-GBM queue, survival analysis showed that fatty acid metabolic process MT had significantly longer OS time than fatty acid metabolic process WT [**Figure 2C**; P = 0.033; Hazard Ratio (HR) = 0.4; 95% CI: 0.22 - 0.75]. We also found that the mutation status of the fatty acid metabolic process can predict other immune queues. For example, in ICI-Bladder cancer, survival analysis showed that fatty acid metabolic process MT resulted in significantly longer OS time than fatty acid metabolic process WT (**Figure 2D**; P = 0.019; HR = 0.5; 95% CI: 0.31 – 0.81). In TCGA-GBM, the mutation state of fatty acid metabolic process has nothing to do with the prognosis state of GBM (**Figure 2E**; P > 0.05). Similarly, in Jonsson-GBM, the OS time and progression-free survival (PFS) time of patients had nothing to do with the mutation state of the fatty acid metabolic process (**Figures 2F, G**).

### The Mutation State of the Fatty Acid Metabolic Process and Immune Cells

**Figure 3:**
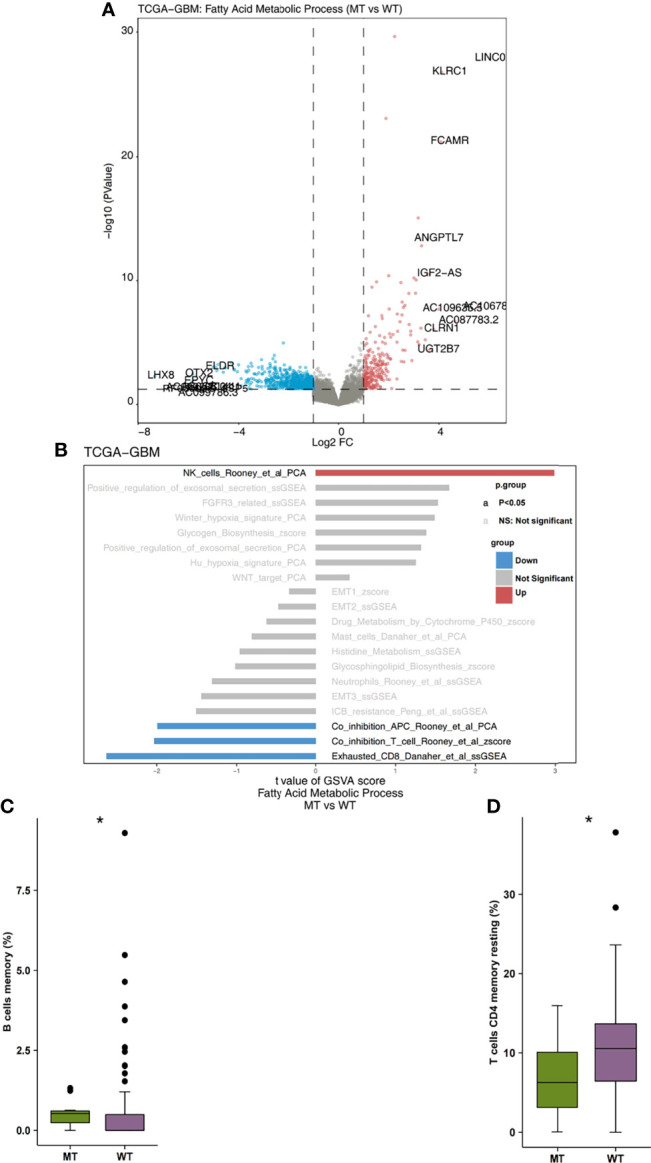
**(A)** The volcano plot of the differentially expressed genes in the TCGA-GBM queues. The red dot represents the significantly up-regulated genes (P < 0.05; log2FC > 1), while the blue dot represents the significantly down-regulated genes (P < 0.05; log2FC < -1). **(B)** The bar plot representing the up-regulated and down-regulated immune cells scores estimated by the ssGSEA method. The red bar represents the significantly up-regulated immune cells in the fatty acid metabolic process of the MT group, while the blue bar represents the significantly down-regulated immune cells in the fatty acid metabolic process of the MT group. The grey bar represents that there are no significantly different immune cells or pathways. **(C)** A comparison of the scores of the memory B cells and the fatty acid metabolic process of the MT and WT groups. **(D)** A comparison of the scores of the resting memory CD4+ T cells CD4 and the fatty acid metabolic processes of the MT and WT groups. MT, mutant-type; WT, wild-type; ssGSEA, Single sample Gene Set Enrichment analysis. *P < 0.05.

Firstly, we analyzed the differences in expression data between fatty acid metabolic process MT and WT. In **Figure 3A**, the genes that were significantly up-regulated and significantly down-regulated in the WT and MT groups are displayed in red and blue, respectively (P < 0.05; |log2FC| > 1). Based on immune-related genes and the ssGSEA algorithm, we found that the fraction of NK cells in the MT group was significantly higher than that in WT group. On the other hand, we also found that the level of depletion among the CD8+T cells, the scores of co-inhibition antigen-presenting cells (APC), and the number of co-inhibition T cells in the MT group were significantly lower than those in WT group (**Figure 3B**; P < 0.05). In addition, using the CIBEROSRT algorithm to evaluate the expression data of patients, we found that the proportion of memory B cells in the MT group was significantly higher than that in the WT group (**Figure 3C**; P < 0.05). On the other hand, the relative ratio of resting memory CD4+ T cells in the MT group was significantly higher than that in WT group (**Figure 3D**; P < 0.05).

### The Mutation State and Immunogenicity of the Fatty Acid Metabolic Process

**Figure 4:**
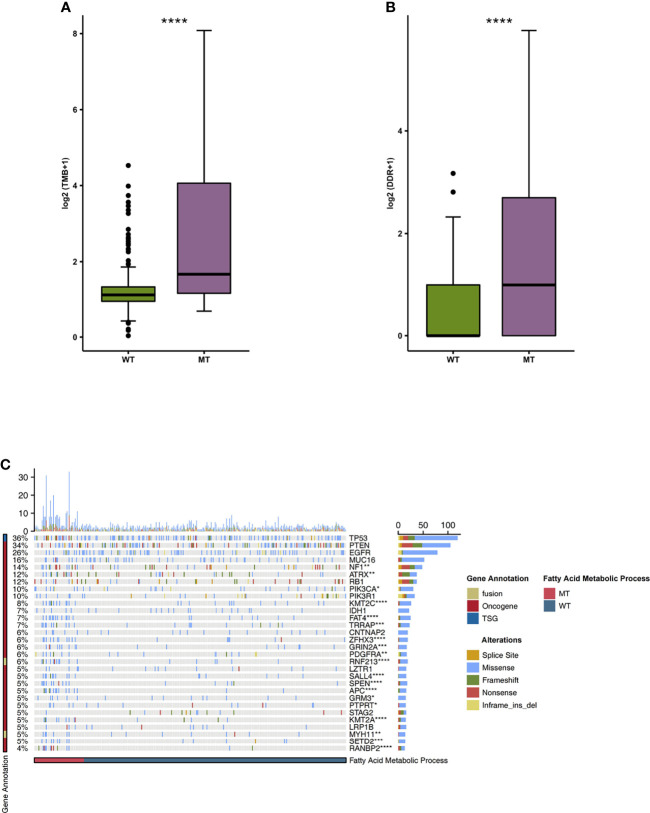
**(A)** A comparison of the TMB levels of the fatty acid metabolic processes of the MT and WT groups. **(B)** A comparison of the mutation counts of DDR signaling of the fatty acid metabolic processes of the MT and WT groups. **(C)** The mutational landscape of the top 30 mutated driver genes in the fatty acid metabolic processes of the MT and WT groups. MT, mutant-type; WT, wild-type; TMB, Tumor mutation burdens; DDR, DNA damage repair. *P < 0.05; **P < 0.01; ***P < 0.001; ****P < 0.0001.

We began by analyzing the relationship between TMB and the mutation state of the fatty acid metabolic process. We found that the level of TMB in the MT group was significantly higher than that in the WT group (**Figure 4A**; P < 0.05). In addition, compared with the WT group, the MT group had a high number of gene mutations in the DDR pathway (**Figure 4B**). We also compared the difference between MT and WT in driving mutations with the top 30 mutation frequencies in the GBM cohort (**Figure 4C**). We found that most of the driving mutations with significant difference belonged to oncogenes, including NF1 (25.49% vs 9.60%), ATRX (23.53% vs 7.62%), PIK3CA (17.65% vs 7.62%), KMT2C (23.53% vs 3.97%), FAT4 (25.49% vs 2.65%), TRRAP (19.61% vs 3.64%), ZFHX3 (21.57% vs 2.32%), GRIN2A (17.65% vs 2.649%), PDGFRA (13.73% vs 3.31%), SALL4 (19.61% vs 1.99%), SPEN (19.61% vs 1.99%), APC (25.49% vs 0.66%), GRM3 (11.76% vs 2.98%), PTPRT (9.80% vs 3.31%), KMT2A (17.65% vs 1.66%), SETD2 (15.69% vs 1.99%), and RANBP2 (21.57% vs 0.66%). We also compared the mutual exclusion and co-occurrence of the driver mutations with the top 30 mutation frequencies in both the MT group and WT group (**Figures S1A, B**).

### The Mutation Status of the Fatty Acid Metabolic Process and Immune-Related Pathways

**Figure 5:**
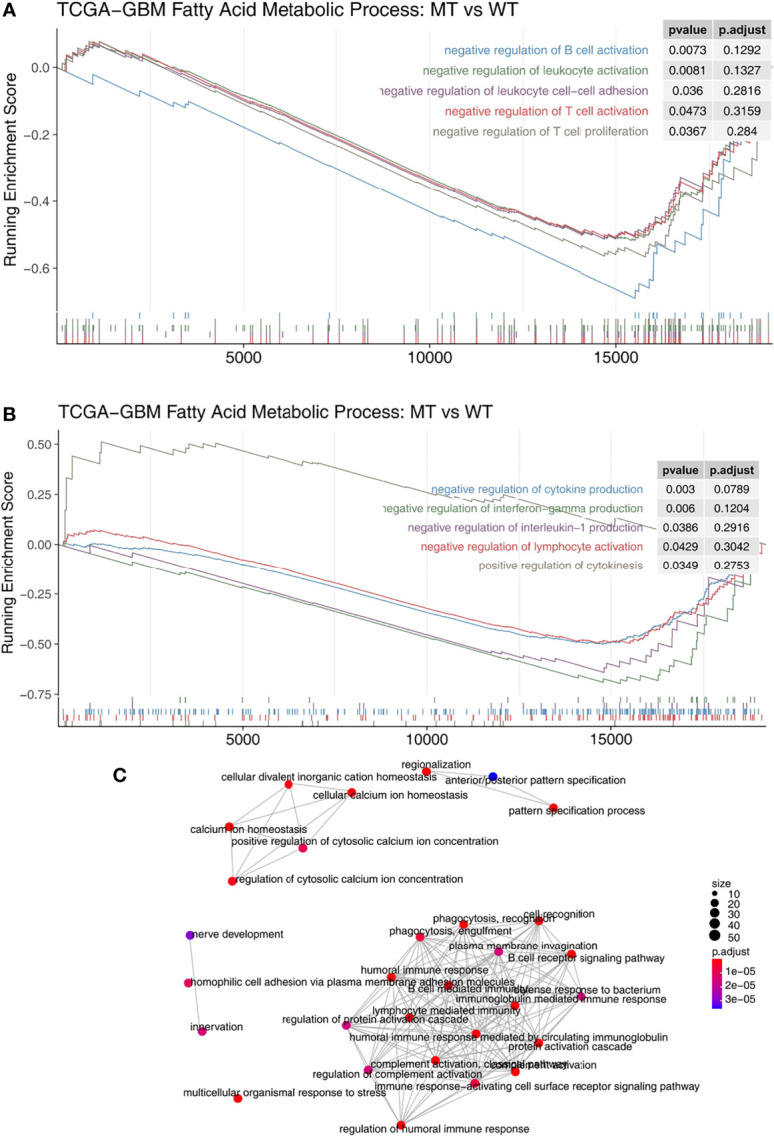
**(A)** The gene sets enrichment analysis showed that the enrichment scores of the immune cells were significantly up-regulated in the fatty acid metabolic process of the MT group. **(B)** The gene sets enrichment analysis showed that the enrichment scores of cytokine production were significantly up-regulated in the fatty acid metabolic process of the MT group. **(C)** The emapplot representing the connection between the immune-related signaling pathways. MT, mutant-type.

We used the GSEA algorithm to analyze the enrichment scores and p-values of the MT group and the WT group in combination with the expression data of GBM patients. We found that compared with the WT group, MT-GBM patients had significantly increased activity in their immune cell activation pathways (**Figure 5A**), such as B cell activation, lymphocyte activation, T cell activation, T cell proliferation, and lymphocyte adhesion. In addition, the immune microenvironment of the MT group was significantly enriched in the inflammatory cytokine secretion pathway (**Figure 5B**), as exhibited by increased IFN-γ and IL-1 secretion and production. The network diagram of enrichment analysis showed that the interaction between the immune activation pathways was very similar (**Figure 5C**).

### The Mutation Status and Drug Sensitivity of the Fatty Acid Metabolic Process

**Figure 6:**
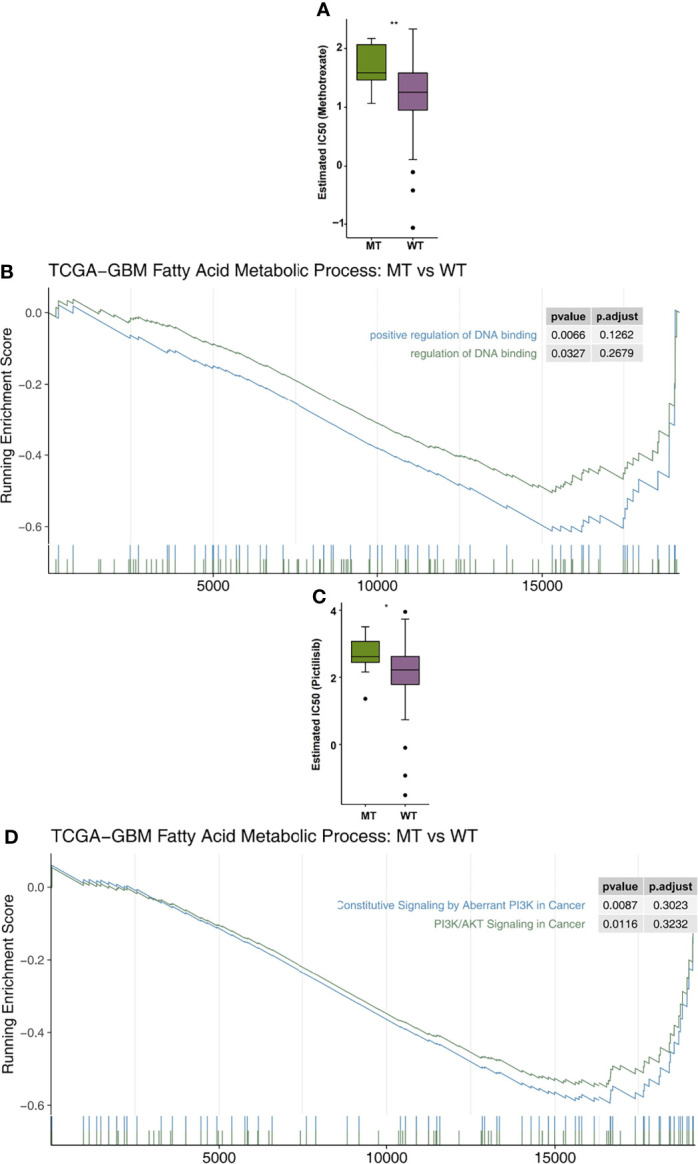
**(A)** A comparison of the estimated IC50 values of methotrexate in the fatty acid metabolic processes of the MT and WT groups. **(B)** The results of the DNA binding signaling GSEA. **(C)** A comparison of the estimated of IC50 values of pictilisib in the fatty acid metabolic processes of the MT and WT groups. **(D)** The GSEA results of PI3K/AKT signaling. MT, mutant-type; WT, wild-type; IC50, half maximal inhibitory concentration; GSEA, Gene Set Enrichment analysis. *P < 0.05; **P < 0.01.

In order to explore the differences in drug sensitivity between the MT group and WT group, the pRRophetic algorithm was used to estimate the drug sensitivity based on each patient’s expression data. We found that the IC50 value of methotrexate in relation to the DNA replication mechanism in the MT group was significantly higher than that in the WT group (**Figure 6A**). GSEA showed that the enrichment of DNA binding related pathways in the MT group was significantly lower than that in the WT group (**Figure 6B**). In addition, we also found that the sensitivity of MT-GBM patients to pictilisib, a PI3K/Akt inhibitor, was significantly lower than that of the WT-GBM patients (**Figure 6C**). GSEA also showed that the enrichment fraction of the PI3K/Akt pathway in the MT group was significantly lower than that in the WT group (**Figure 6D**).

## Discussion

In this study, univariate and multivariate Cox regression analyses showed that fatty acid metabolic process MT may be an independent prognostic factor for GBM patients receiving ICI treatment. In addition, Kaplan Meier survival analysis showed that the MT group had significantly longer OS time than the WT group. The results of immune microenvironment analysis showed that the MT group had an inflammatory immune microenvironment that manifested as significantly up-regulated NKT cells and significantly down-regulated exhausted CD8+ T cells. The results showed that the activation of immune cells, as well as the secretion and production pathways of inflammatory molecules, were significantly activated in the MT group. At the same time, the MT group displayed a significant increase in immunogenicity compared to the WT group.

The higher immunogenicity of patients with fatty acid metabolic process MT is potentially the mechanism behind patients’ better prognoses post-ICI treatment. Patients with fatty acid metabolic process MT not only had higher TMB, but also a higher number of gene mutations in their DDR pathway, which improved their immunogenicity. Due to gene fusion, deletion mutation, and point mutation of tumor genes, new abnormal proteins, which are new antigens encoded by the mutant genes of tumor cells, are produced (31). These new tumor antigens can not only enhance the immune response of T cells, but also increase the content of new antigen-specific T cells, thus improving the immune system’s ability to identify tumors (32). Studies have shown that the mutation of the DDR pathway will result in the production of higher TMB and neoantigen loads, thus significantly improving the effectiveness of ICI treatment (33–35).

An inflammatory immune microenvironment also results in greater effectiveness for ICIs treatment in patients with fatty acid metabolic process. The MT group showed a significant increase in activated immune cells such as NK cells and a significant decreased in depleted CD8+ T cells. In these groups, we found that NKT cells killed tumor cells in three ways and thus played an important anti-tumor role (1): in the Fas/FasL, perforin, and granzyme B pathways, they played a cytotoxic role and directly kill tumor cells (36) (2); they regulate the recruitment and function of other immune cells by secreting cytokines, thus indirectly playing an anti-tumor role (37) (3); NKT cells changed the immunosuppression level in the immune microenvironment, which resulted in anti-tumor activities (38). In addition, when CD8+T cells bind to antigens from dendritic cells, cytotoxic CD8+T cells can be produced. Under the induction of chemokines secreted by the dendritic cells CXCL9 and CXCL10, activated cytotoxic CD8+T cells were able to migrate to the inflammatory environment through CXCR3 expression (39). In the immune microenvironment, IL-1 helped to establish a pro-inflammatory environment by inducing pro-inflammatory cytokines and chemokines (40). IFN-γ played a central role in immunity by inducing MHCI molecules, Fas/FasL, and inducing immune proteasome-related factors (41). In the IFN-γ environment, the main cytokines secreted by activated CD8+T cells significantly increased the expression of PD-L1 (42).

This study has some limitations. Firstly, the number and sample size of clinical studies of glioma patients treated with immune checkpoint inhibitors in the database are currently small, so we hope to recruit more GBM patients who have received ICI therapy in the future to further verify the conclusions of this study. Second, few animal or cell experiments have been done on the subject. Third, the ICI-GBM cohort contained only the mutation data and clinical prognosis data of targeted sequencing, so there may have been potential bias when calculating the number of gene mutations in the fatty acid metabolic process pathway.

## Conclusions

In this study, we found that fatty acid metabolic process MT may be used as an independent predictor of the efficacy of ICI treatment in GBM patients, which will allow doctors to better screen out the dominant population receiving immunotherapy in the future. Fatty acid metabolic process MT is related to higher immunogenicity, a significant increase in the quantity of activated immune cells, and improvement to the immune microenvironment.

## Data Availability Statement

The datasets presented in this study can be found in online repositories. The names of the repository/repositories and accession number(s) can be found in the article/**Supplementary Material**.

## Ethics Statement

Ethical review and approval was not required for the study on human participants in accordance with the local legislation and institutional requirements. Written informed consent for participation was not required for this study in accordance with the national legislation and the institutional requirements.

## Author Contributions

Conceptualization, BL and ZQ. Formal analysis, RL, WL, QH, LW. Visualization, RL, QL. Writing–original draft, RL, WL, QH, LW, FZ, BL and ZQ. Writing–review & editing, RL, WL, QH, LW, FZ, BL and ZQ. All authors read and approved the final manuscript.

## Funding

This work was supported by the Science and Technology Project of Education Department of Jiangxi Province (180805), the Science and Technology Project of Health and Family Planning Commission of Jiangxi Province (20195363), the CSCO-Haosen Oncology Research Fund (Y-HS2019/2-015), and the Science and Technology Project of Ganzhou City (GZ2021SF002).

## Conflict of Interest

The authors declare that the research was conducted in the absence of any commercial or financial relationships that could be construed as a potential conflict of interest.

## Publisher’s Note

All claims expressed in this article are solely those of the authors and do not necessarily represent those of their affiliated organizations, or those of the publisher, the editors and the reviewers. Any product that may be evaluated in this article, or claim that may be made by its manufacturer, is not guaranteed or endorsed by the publisher.

## Supplementary Material

Supplementary Figure 1 The co-exclusivity and co-occurrence of top 30 mutated driver genes in the fatty acid metabolic processes of the WT **(A)** and MT **(B)** groups. MT, mutant-type; WT, wild-type.

Supplementary Table 1 The clinical characteristics of the ICI-GBM cohort.
