# Supplementary figures and images for: Fatty Acid Metabolic Signaling Pathway Alternation Predict Prognosis of Immune Checkpoint Inhibitors in Glioblastoma
Source: Front Immunol. 2022 Feb 18;13:819515. doi: 10.3389/fimmu.2022.819515 (PMC8894256; doi:10.3389/fimmu.2022.819515)

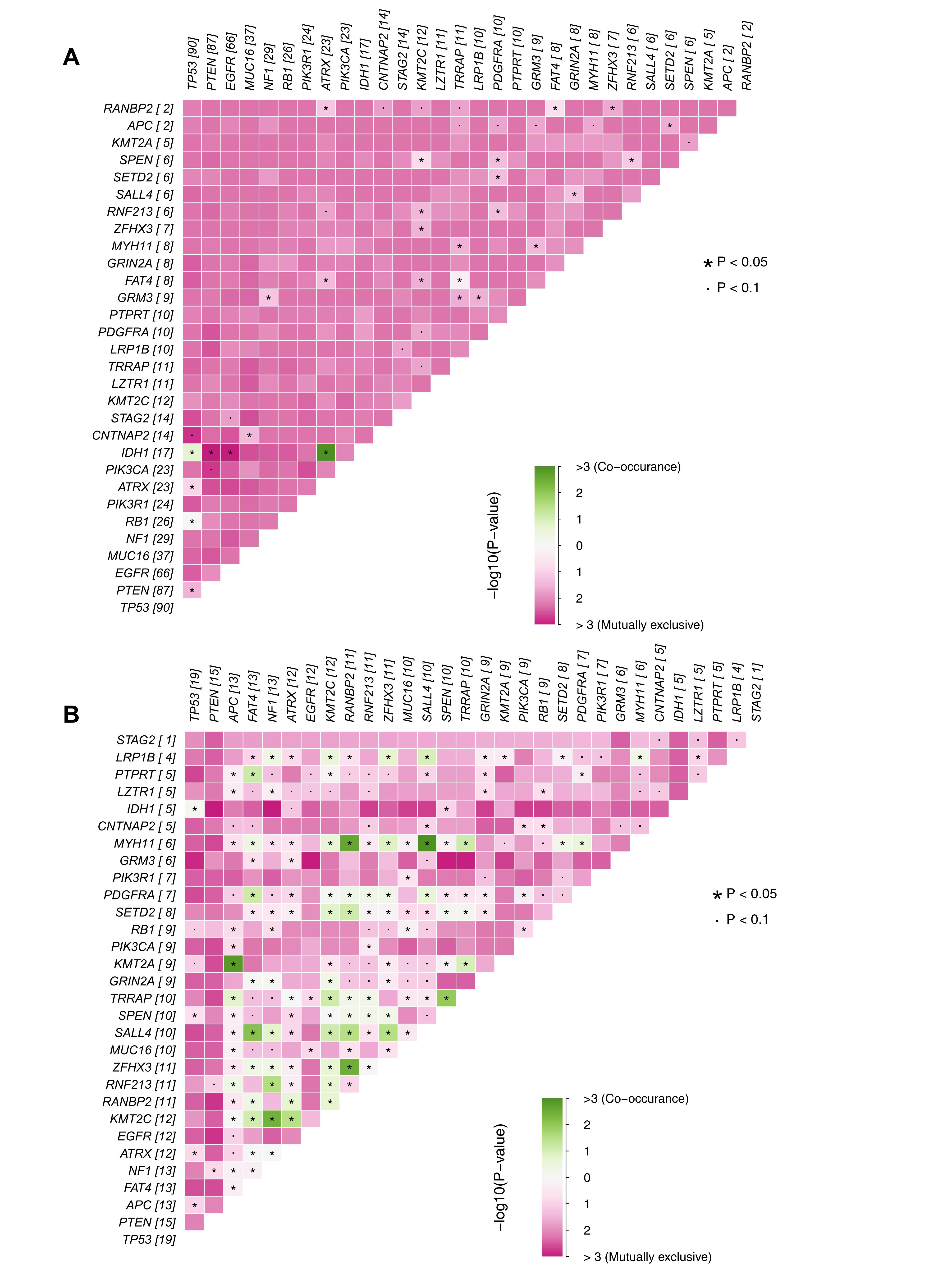

Supplement: Supplementary Figure 1 — The co-exclusivity and co-occurrence of top 30 mutated driver genes in the fatty acid metabolic processes of the WT (A) and MT (B) groups. MT, mutant-type; WT, wild-type. [file Image_1.jpeg]
