# Supplementary material for: Fatty Acid Metabolic Signaling Pathway Alternation Predict Prognosis of Immune Checkpoint Inhibitors in Glioblastoma
Source: Front Immunol. 2022 Feb 18;13:819515. doi: 10.3389/fimmu.2022.819515 (PMC8894256; doi:10.3389/fimmu.2022.819515)
Supplement: Supplementary Table 1 — The clinical characteristics of the ICI-GBM cohort. [file Table_1.pdf]

	Fatty Acid Metabolic Process-MT (N=12)	Fatty Acid Metabolic Process-WT (N=103)	Overall (N=115)
**Drug Type**			
Combination	0 (0%)	3 (2.9%)	3 (2.6%)
PD-1/PDL-1	12 (100%)	100 (97.1%)	112 (97.4%)
**Age**			
Mean (SD)	51.4 (16.7)	51.5 (14.4)	51.5 (14.6)
Median [Min, Max]	54.5 [25.0, 80.0]	54.0 [15.0, 77.0]	54.0 [15.0, 80.0]
**TMB**			
Mean (SD)	25.7 (33.5)	5.86 (7.39)	7.93 (13.9)
Median [Min, Max]	5.10 [2.23, 100]	3.94 [0.980, 51.2]	4.39 [0.980, 100]
